# The Psychological Impact of Experiencing Sexual Abuse Revictimization by a Different Perpetrator in Childhood

**DOI:** 10.3390/children12081070

**Published:** 2025-08-14

**Authors:** Elizabeth L. Jeglic, Georgia M. Winters, Benjamin N. Johnson, Emma Fisher

**Affiliations:** 1 Department of Psychology, John Jay College of Criminal Justice, New York, NY 10019, USA; 2 School of Psychology and Counseling, Fairleigh Dickinson University, Teaneck, NJ 07666, USA; gwinters@fdu.edu (G.M.W.); b.johnson2@fdu.edu (B.N.J.);

**Keywords:** child sexual abuse, multiple, single, perpetrator, mental health, depression, PTSD, consequences, revictimization

## Abstract

Background/Objectives: Research has shown that those who experience childhood sexual abuse (CSA) are at increased risk of subsequent sexual revictimization. Multiple sexual victimizations can lead to higher rates of depression, anxiety, trauma, and suicidality. Prior research has yielded varying definitions of revictimization, including only accounting for revictimization that occurred in adulthood or multiple CSA episodes by the same perpetrator, or it has broadly assessed maltreatment without a specific focus on CSA. This study examined mental health outcomes in survivors of CSA who experienced sexual revictimization in childhood from a different perpetrator, comparing their mental health outcomes (i.e., depression, suicidal ideation, post-traumatic stress disorder (PSTD), hopelessness, guilt, and shame) to those who reported CSA by one perpetrator. Methods: Adult survivors of CSA (*n* = 627) completed an online survey describing their CSA experience, whether they experienced CSA by one or multiple perpetrators in childhood, and a series of mental health questionnaires. Results: Almost half of the sample reported CSA by more than one perpetrator in childhood (*n* = 267; 42.58%). Survivors who reported multiple CSA perpetrators reported significantly higher levels of depression, suicidal thoughts, PTSD, hopelessness, shame, and some facets of guilt in adulthood compared to those who reported CSA by a single perpetrator. Conclusions: Experiencing CSA by multiple perpetrators in childhood may lead to more negative mental health outcomes in adulthood. The findings emphasize the importance of early identification and intervention for individuals who experienced CSA.

## 1. Introduction

Childhood sexual abuse (CSA) affects approximately 2.5–7.8% of males and 11.4–25% of females in the United States [1,2,3,4]. CSA leads to serious long-term consequences for survivors, their families, and the broader community. Survivors commonly experience depression, anxiety, hopelessness, isolation, low self-esteem, substance abuse, suicidal behavior, and trauma symptoms [5,6,7,8,9]. Further, research shows that 17–39% of CSA survivors experience sexual revictimization during childhood or adolescence [10].

Multiple CSA victimizations can increase the risk of severe psychological and behavioral problems [5,11,12,13]. However, research to date lacks a clear definition of sexual revictimization, often relying on retrospective studies spanning into adulthood [14,15], assessing multiple occurrences of CSA as opposed to multiple separate perpetrators, and/or measuring a variety of adverse childhood experiences (such as physical abuse and neglect) with CSA included [15,16]. As prevention, treatment, and legal decision-making can be impacted by information about CSA revictimization, this study focused specifically on sexual revictimization that occurred during childhood to compare mental health outcomes among adult survivors of CSA who reported single versus multiple perpetrators in childhood.

### 1.1. CSA and Mental Health

In the U.S., psychiatric disorders are more common in individuals with a CSA history, even after controlling for other adverse childhood experiences [4]. Molnar and colleagues, using a national census survey, found that individuals with a history of CSA were more likely to have a PTSD diagnosis than those without a history of CSA [4]. Women with a CSA history were twice as likely to report depression (39.2%) as those without (19.2%). Men with CSA history reported nearly triple the rate (30.3%) compared to men without CSA history (11.4%). Further, survivors of CSA have described intense hopelessness and feelings of shame or guilt because of the CSA, as well as engaging in suicide attempts/non-suicidal injurious behaviors [17,18].

An umbrella review of over four million adults found that CSA survivors were 2.2 to 3.3 times more likely to be diagnosed with psychiatric disorders, including eating disorders, PTSD, depression, anxiety, borderline personality disorder, and conversion disorder. They were also nearly twice as likely to have attempted suicide [7]. Finally, a five-decade longitudinal population-based study found that, even after controlling for confounding factors, individuals who self-reported child sexual abuse experienced a wide range of negative long-term outcomes—including significantly higher rates of internalizing and externalizing disorders, thought disorders, suicide attempts, and health risk behaviors [6].

### 1.2. Revictimization

Those who experience CSA at least once are at significantly increased risk of subsequent sexual revictimization, with rates ranging between 1.9 and 11.7 times [7,8,19]. However, there is a wide range of definitions of revictimization, with some studies broadly defining it as experiencing sexual abuse more than once, which could include multiple victimizations by the same perpetrator [20], while other studies include sexual revictimization that occurs only in adulthood [7,12,21]. A recent meta-analysis of rates of sexual victimization found that 47.9% of the 12,252 individuals who reported CSA across 80 studies reported revictimization. To be included in the meta-analysis, the studies needed to have measured sexual victimization at two distinct time points, with the first time point occurring either in childhood or adolescence, but there were no reference age criteria for the subsequent time period [22]. Based on this definition, it is not possible to ascertain whether the sexual victimization at the second time point pertained to the same perpetrator (multiple instances of CSA), multiple perpetrators in childhood, and/or revictimization in adulthood.

One of the few studies that examined revictimization in childhood alone was conducted by Boney-McCoy and Finkelhor [19]. They conducted telephone surveys of a random sample of American children aged 10–16 and asked them questions about their victimization history. Of the 2000 children in the sample, 132 reported CSA in the past year, and of those, 46 (34.8%) reported prior sexual victimization. However, it was unclear whether the revictimization was from the same or a different perpetrator. Further, they defined CSA broadly to include behaviors such as being propositioned by an older, unrelated individual; being propositioned by a parent or witnessing an exhibitionist; and contact behaviors such as fondling above clothes and genital penetration. Indeed, only 33% of the original sample of those who experienced CSA reported contact sexual abuse [19]. Such findings highlight that there remains ambiguity as to how the term “revictimization” is being used in the field.

### 1.3. Revictimization and Mental Health

Earlier studies found that adults who experienced both CSA and later sexual revictimization reported greater psychological distress than those abused in just one time period or not at all [23,24]. For example, Balsam et al. (2011) surveyed adults about their abuse experiences [12]. They found that individuals who reported revictimization (defined as experiencing both CSA and adult sexual abuse) reported significantly higher levels psychological distress, suicidality, alcohol and drug use, and self-harm compared to those with only one type of victimization or no victimization. Similarly, Lau and Kristensen (2010) found that in a sample of 161 women who reported a history of CSA, those who also reported adult sexual abuse (36%) had experienced more suicide attempts, psychological distress, and cognitive distortions related to fear, being scared or shy, and mistrust [25]. In one of the only studies that only examined revictimization in childhood, Boney-McCoy and Finkelhor (1995) found that those children who reported revictimization (definition described earlier) reported significantly more symptoms of PTSD [19]. It should be noted that in this study, participants were not asked if their PTSD symptoms pertained to the CSA event specifically.

### 1.4. The Present Study

Overall, research has shown that those who experience sexual victimization once are at increased risk of subsequent revictimization [14,15,16,26] and that revictimization is associated with more serious negative mental health consequences [5,11,12,13]. However, to our knowledge, no study to date has examined sexual revictimization by a different perpetrator occurring solely in childhood (under the age of 18). For this reason, the present study aims to examine mental health outcomes in survivors of CSA who experienced sexual revictimization in childhood from a different perpetrator, comparing their self-reported mental health outcomes (e.g., depression, suicidality, PSTD, hopelessness, guilt, and shame) to those who reported CSA by one perpetrator. We hypothesize that those who report experiencing sexual abuse by more than one perpetrator in childhood will report higher levels of depression, suicidality, PTSD, hopelessness, guilt, and shame during adulthood compared to those who report victimization by a single perpetrator.

## 2. Methods and Participants

Data for the current study was drawn from a larger investigation into sexual grooming behaviors among adult survivors of CSA. Participants were recruited through Prolific, an online research platform which yields more diverse and better-quality responses than other comparable online survey sites [27]. Eligibility for participation was ascertained via a brief screening survey, for which participants received USD 0.13 compensation. Inclusion criteria included (1) a history of unwanted sexual contact prior to age 18; (2) currently being 18 years of age or older; (3) fluency in reading and writing English; and (4) current residence in the United States. Those who met these inclusion criteria were invited to complete a 30 min survey about their CSA experiences. Participants who completed the full survey received USD 5.00 and were provided with a debriefing form that included the study’s purpose, the investigator’s contact information, and mental health resources. All procedures received approval from the Institutional Review Boards of the primary investigators’ institutions. Of the 734 individuals who provided informed consent, 53 were excluded due to incomplete responses or failing two of the three attention check questions and 54 were excluded because they did not answer the perpetrator questions—the key variable being studied in this investigation. The final sample included 627 adults who reported experiencing CSA by one or more perpetrators in childhood.

Participant demographics are presented in the overall sample column in Table 1. Participants were 35 years old (*M* = 34.8, *SD* = 12.1; range 18–77), primarily female sex at birth (*n* = 464, 74.0%), self-identified their gender identity as women (*n* = 433, 69.1%), and their race/ethnicity primarily White/of European descent (*n* = 449, 71.6%).

### Measures

**Number of perpetrators**: Participants were asked whether they had experienced CSA and, if so, whether it involved more than one perpetrator during childhood. Those who answered yes were then asked to specify the number (2, 3, or 4+). Individuals who reported more than one perpetrator were classified as the multiple-perpetrator group.

**Beck Depression Inventory**—**Second Edition (BDI-II):** The BDI-II is a 21-item self-report measure assessing depressive symptoms over the past two weeks, a common long-term consequence of CSA [7]. Each item has four response options indicating increasing severity. Total scores are interpreted as follows: 0–9 (minimal), 10–19 (mild), 20–30 (moderate to severe), and 31+ (severe) [28]. The BDI-II has demonstrated strong reliability and validity [29]; internal consistency in this sample was excellent (α = 0.95).

**Suicidal ideation**: Item 9, which assesses suicidal ideation, was analyzed separately. Responses range from 0 (“I do not have any thoughts of killing myself”) to 3 (“I would kill myself if I had the chance”), with any score above 0 indicating suicidal ideation.

**PTSD Checklist for DSM-5 (PCL-5)**: The PCL-5 is a 20-item self-report measure assessing PTSD symptom severity over the past month, based on DSM-5 criteria [30]. Items are rated on a five-point Likert scale from 0 (Not at all) to 4 (Extremely), with total scores of 31–33 suggesting probable PTSD. The measure has shown strong psychometric properties, including internal consistency, test–retest reliability, and both convergent and discriminant validity [30]. In this sample, internal consistency was excellent (Cronbach’s α = 0.95).

**The Beck Hopelessness Scale (BHS)**: The BHS is a 20-item true–false self-report measure developed to assess the extent to which an individual’s thoughts are characterized by negative expectations about the future [31]. Higher scores on the BHS have been associated with an increased risk of future suicide attempts [32], and thus this measure was administered to obtain a more comprehensive evaluation of mood disturbance. In the current sample, the scale demonstrated excellent internal consistency (Cronbach’s α = 0.96).

**Trauma-Related Guilt Inventory (TRGI):** The TRGI is a 32-item self-report measure assessing cognitive and emotional aspects of guilt related to a traumatic event [33]. Participants rate each item on a five-point Likert scale from “Never True” to “Extremely True.” The measure includes six subscales: global, distress, guilt cognitions, hindsight/responsibility, wrongdoing, and lack of justification. The TRGI has demonstrated high internal consistency, good test–retest reliability, and strong convergent validity [33]. In this study, participants completed the TRGI in reference to their CSA experience. Internal consistency for the total score was strong (α = 0.91), with acceptable reliability across subscales (α = 0.79–0.91).

**Trauma-Related Shame Inventory (TRSI):** The TRSI is a 24-item self-report scale measuring trauma-related shame [34]. Respondents rate items on a four-point Likert scale from 0 (Not true of me) to 3 (Completely true of me), with higher scores indicating greater shame. The scale includes two subscales: Internalizing (e.g., shame, disgust, feelings of unworthiness) and externalizing (e.g., perceived judgment or rejection by others). The TRSI has demonstrated strong construct validity and internal consistency [34]. In this study, participants completed the TRSI regarding their CSA experiences. Internal consistency was excellent for the total scale (α = 0.98) and both subscales (α = 0.96).

## 3. Results

Participant characteristics by group (single vs. multiple perpetrator) are presented in Table 1. Of the participants, 360 (57.42%) individuals reported victimization by one perpetrator in childhood, with 267 (42.58%) individuals reporting multiple perpetrators of CSA in childhood. Of those who reported more than one perpetrator, 169 (26.95%) reported two perpetrators, 66 (10.53%) reported three (5.10%) perpetrators, and 32 reported more than four perpetrators.

Significant differences were found between groups regarding participant sex (χ^2^ = 26.85, *p* < 0.01), with males being less likely to experience CSA from multiple perpetrators compared to females, and gender identity (χ^2^ = 23.52, *p* < 0.01), with those identifying as “other” (non-binary, transgender) more likely to report CSA by multiple perpetrators. No significant differences in the rates of multiple-perpetrator victimization were found based on participant race/ethnicity or age.

Means, standard deviations, and effect sizes for the mental health sales and subscales by group are presented in Table 2. Due to unequal sample sizes, Welch’s two-sample *t*-tests were used to compare differences. As depicted in Table 2, significant differences between groups were found for the BDI-II, Item 9 of the BDI-II (suicide), the BHS, the PCL-5, the TGRI global and distress subscales, and the TRSI. In all cases, the means were significantly higher (denoting increased symptomatology) for those who reported victimization by multiple perpetrators versus a single perpetrator. No significant differences were found between the groups for the TGRI cognitive, hindsight, justification, and wrongdoing subscales.

## 4. Discussion

This is one of the first studies to examine differences in multiple self-reported mental health outcomes in adulthood among survivors of CSA between those who had experienced CSA by one versus multiple perpetrators before the age of 18. Overall, we found that experiencing CSA by more than one perpetrator was not uncommon in our sample, with close to half of the sample (42.58%) reporting more than one perpetrator of CSA in childhood. Further, those who reported CSA by multiple perpetrators before the age of 18 also reported more symptoms of depression, suicidal thoughts, PTSD, hopelessness, and some facets of guilt and shame compared to those who reported CSA by a single perpetrator.

Similarly to past research, we found that those who reported revictimization reported more psychological distress than those who reported CSA by one perpetrator [23,24,35]. However, most past research has examined multiple instances of CSA by one perpetrator or revictimization by a different perpetrator in adulthood—this is the first study to explicitly examine CSA revictimization by a different perpetrator in childhood. Studies have consistently found that those who experience CSA in general, and specifically those who experience sexual revictimization, report elevated rates of trauma related symptoms/PTSD [7]. In this study, only participants who reported CSA by multiple perpetrators in childhood met the cutoff for likely PTSD on the PCL-5, whereas those reporting CSA by a single perpetrator did not. Several theories attempt to explain the link between childhood sexual abuse (CSA) and later revictimization. Some studies postulate that PTSD acts as a mediator for revictimization. For example, Ullman and colleagues (2009) found that PTSD numbing symptoms experienced following CSA directly predicted revictimization in a sample of 555 women who experienced CSA before the age of 14 and sexual abuse after the age of 14. Other PTSD symptoms—such as reexperiencing, avoidance, and arousal following CSA—were associated with problem drinking, which in turn elevated the risk of being revictimized. Other studies focus more on the cumulative effect of multiple traumas, suggesting that revictimization impacts coping, thus increasing the severity of PTSD symptoms [36].

Depression is a well-established long-term consequence of CSA [6,7], and in the current study, it was found to be significantly more severe among individuals who experienced abuse by multiple perpetrators in childhood compared to those abused by a single perpetrator. Suicidal ideation and feelings of hopelessness were also more prevalent in the multiple-perpetrator group. The relationship between depression and revictimization may be bidirectional, with initial depressive symptoms following CSA increasing vulnerability to further victimization, which in turn exacerbates depressive symptoms [27]. Consequently, individuals experiencing heightened negative affect, suicidal thoughts, and hopelessness after CSA may be at elevated risk of subsequent abuse. These findings are consistent with both cognitive theories of depression and cumulative risk frameworks, which posit that repeated exposure to adverse events—such as abuse by multiple perpetrators—can contribute to increasingly negative internalized beliefs about the self, others, and the future [31,32]. It is also important to highlight that, although the effect size was small, the observed differences in suicidal ideation between groups may carry meaningful clinical implications. Prior research has shown that individuals with a history of CSA are nearly twice as likely to attempt suicide compared to those without such a history [7]. Given the established link between CSA-related shame and suicidality [37], the elevated suicidal thoughts reported by those with multiple-perpetrator experiences underscore the urgent need for targeted suicide prevention efforts tailored to revictimized populations.

Interestingly, we found that individuals who experienced CSA by multiple perpetrators reported significantly more shame but generally no more guilt than those who were victimized by a single perpetrator in childhood. In the CSA literature, the distinction between shame and guilt is often blurred, as these terms are frequently used interchangeably [38]. However, guilt typically refers to a negative evaluation of one’s behavior, while shame involves a more global condemnation of the self (e.g., “I am a bad person”) [39]. For many survivors, this distinction is not always apparent, and both emotions can contribute to self-loathing and self-stigmatization following CSA. As such, attempts to separate shame and guilt can be conceptually difficult and may also limit therapeutic effectiveness [40].

In this study, the TRGI assessed guilt-related responses focused on behavior following a traumatic event, while the TRSI measured emotional reactions—particularly shame—associated with the participant’s CSA experience. It has been proposed that guilt may operate as a surface-level emotion that masks deeper, more pervasive feelings of shame [15]. This may help explain why survivors of multi-perpetrator CSA reported significantly higher scores on the TRGI global guilt and distress subscales but not on the TRGI subscales targeting more cognitive dimensions of guilt, such as guilt cognitions, hindsight bias/responsibility, wrongdoing, and lack of justification. This pattern, which is characterized by elevated emotional but not cognitive guilt symptoms, supports the idea that shame is more closely tied to one’s identity than guilt and is more likely to manifest as intense emotional distress [32,39]. It suggests that individuals abused by multiple perpetrators may carry more internalized shame, even if they do not explicitly endorse greater cognitive self-blame.

## 5. Limitations

The current study is not without limitations. While this is one of the first studies to examine mental health outcomes among adult survivors of CSA who had one versus multiple perpetrators in childhood, we cannot distinguish between those who had multiple perpetrators across time versus those who had multiple perpetrators at the same time. While most CSA involves only one perpetrator at a time [41], there could be instances where there were multiple perpetrators at the same time (e.g., gang rape), which results in more severe mental health outcomes [41] compared to those with only one perpetrator per incident. Additionally, this is a cross-sectional design and thus we cannot conclusively attribute these mental health outcomes and the differences between these outcomes between groups to experiences of CSA alone. Future studies using longitudinal designs and non-abused control groups would be helpful in disentangling this limitation. Additionally, we did not assess socioeconomic status, and we had too few individuals who identified as gender or racial/ethnic minorities to conduct adequately powered and clinically meaningful comparisons. Oversampling for these groups, which may be at increased risk of CSA victimization, would be important for future studies. We also did not assess whether individuals sought treatment following the CSA, which could impact the findings as treatment has been found to decrease symptoms of CSA in adulthood [42]. Finally, while sufficiently large to examine the research question, our sample is limited to those who were registered on the Prolific website and who chose to participate in this study, thereby limiting the generalizability of the findings. Future studies with more population-based sampling could help to overcome this limitation.

## 6. Implications

### 6.1. Treatment Implications

While it has already been well established that there are numerous long-term mental health consequences for those who have experienced CSA in general [6,7], it appears that those who have experienced CSA by multiple perpetrators in childhood also reported more severe current mental health problems, which has implications for treatment. Without longitudinal data one cannot solve the chicken–egg problem as to whether the mental health problems preceded the multiple-perpetrator CSA or are in fact a result of it. Regardless, the participants in this study who experienced multi-perpetrator victimization in childhood reported having experienced more psychological distress and specifically more trauma, depression, hopelessness, suicidal ideation, and shame than those who experienced CSA by one perpetrator. This information can inform treatment intensity and targets. While CSA, in general, carries with it feelings of shame for survivors, these feelings are likely exacerbated when there are multiple perpetrators [43]. Blais and Renshaw (2013) found that shame is associated with help-seeking behaviors, such that shame has been shown to hinder access to therapy [35,44]. It has also been found that shame may in fact mediate the relationship between sexual grooming behaviors experienced in CSA and suicidal thinking, thus serving as a potential mechanism for long-term distress [37]. Therefore, targeting feelings of shame related to CSA may be important for CSA survivors in general, as well as specifically for those that experienced revictimization in childhood, to minimize long-term psychological distress. A scoping review of the general psychotherapy literature (not specifically related to CSA) found that treatments that were successful in addressing internalized shame included elements such as psychoeducation, experiential exercises, and techniques to increase social support and emotional expression [45]. A recent systematic review of the psychotherapy literature regarding adult survivors of CSA found only seven studies that met the inclusion criteria, with most of these studies only targeting some of the long-term consequences of CSA, such as PTSD, depression, and/or anxiety. The authors concluded that there is currently little evidence that one form of treatment is better than another but that, overall, adults who seek psychological treatment will experience amelioration in at least one symptom. They noted that Dialectical Behavior Therapy (DBT) was most effective at decreasing anxiety for survivors who had experienced revictimization [42]. For child survivors of CSA and their families, trauma-focused Cognitive Behavioral Therapy (TF-CBT) that actively involves caregivers has shown the most promising outcomes. Given the link between victim vulnerability, including psychopathology, and CSA, it is plausible that interventions that decrease psychological distress could decrease revictimization risk [10]. However, no studies to date have investigated whether treatment can reduce the risk of revictimization by a different perpetrator during childhood [46].

### 6.2. Legal Implications

The findings of this study also have implications in civil legal settings where survivors are required to demonstrate harm that was caused by the abuse. For example, if a survivor of CSA chooses to pursue civil litigation, they must prove that the preponderance of evidence shows that the defendant’s actions caused the harm. However, those that have an existing history of CSA may have difficulty demonstrating that the revictimization caused harm above and beyond the previous victimization. However, these data provide some evidence that those who experience revictimization from a different perpetrator do experience more severe symptoms of PTSD, depression, and feelings of shame and guilt, contributing to increased harm.

## 7. Conclusions

This study found that adult survivors of CSA involving multiple perpetrators reported higher levels of depression, suicidal thoughts, PTSD symptoms, hopelessness, shame, and certain aspects of guilt than those who experienced CSA by a single perpetrator. These findings add to the previous research by suggesting that children who experience multi-perpetrator CSA may experience more internalized shame, leading to a negative view of themselves and resulting in more emotional distress.

These findings also underscore the need for early identification and intervention when CSA is disclosed or suspected. Future research should prioritize developing effective methods to detect early signs of CSA, such as sexual grooming behaviors or changes in children’s behavior, so that timely treatment can be provided. Additionally, further studies are necessary to determine whether treatments like TF-CBT can effectively prevent revictimization and reduce the long-term impacts of CSA.

## Figures and Tables

**Table 1:** Descriptives for demographical characteristics.

Characteristic	Overall Sample (*N* = 627)	Single Victimization (*N* = 360)	Multiple Victimizations (*N* = 267)	Statistic	*p*-Value	Effect Size
	*n* (%)	*n* (%)	*n* (%)	χ^2^	*p*	Cramer’s *V*
*Sex at Birth* ^1^						
Female	464 (74.00)	237 (71.43)	227 (72.99)	**26.85**	**<0.01**	**0.21**
Male	160 (25.52)	120 (28.57)	40 (27.01)			
*Gender Identity*						
Woman	433(68.87)	262 (41.78)	187 (29.82)	**23.52**	**<0.01**	**0.19**
Man	156 (24.50)	117 (25.71)	39 (27.01)			
Other	38 (4.30)	14 (3.88)	24 (8.98)			
*Race/ethnicity*						
White	449 (71.61)	262 (72.77)	187 (70.03)	6.13	0.53	0.1
Black/African American	56 (8.96)	32 (8.88)	24 (3.82)			
Hispanic or Latino	41 (6.54)	20 (5.55)	21 (3.35)			
Asian or Pacific Islander	35 (5.58)	24 (6.67)	11 (1.75)			
Biracial	23 (3.67)	12 (3.33)	11 (1.75)			
Multiracial	18 (2.67)	8 (2.22)	10 (1.59)			
Other	4 (0.64)	2 (0.56)	2 (0.32)			
Prefer Not to Say	1 (0.16)	0 (0.00)	1 (0.16)			

^1^ The sex column does not total 627 as one participant identified their sex at birth as “other” and two preferred not to answer. Bold values indicate statistically significant difference.

**Table 2:** Outcome comparisons by number of perpetrators.

Outcome	Total (*N* = 602)	One Perpetrators (*n* = 350, 58.14%)	More Than One Perpetrator (*n* = 252, 41.86%)	Statistic	*p*-Value	Effect Size
	*M* (*SD*)	*M* (*SD*)	*M* (*SD*)	*Welch’s *t*-test*	*p*	Cohen’s d
BDI-II Total	20.29 (14.85)	17.67 (14.39)	23.85 (14.76)	**−5.21**	**<0.001**	**0.43**
BDI Suicide	0.51 (0.71)	0.46 (0.70)	0.60 (0.74)	**−2.49**	**0.01**	**0.20**
PCL-5 Total	29.46 (19.89)	26.07 (18.51)	33.99 (20.79)	**−4.91**	**<0.001**	**0.41**
BHS	7.53 (6.28)	6.75 (6.22)	8.48 (6.33)	**−3.40**	**<0.001**	**0.28**
TRSI Total	1.79(0.59)	1.68 (0.61)	1.94(0.56)	**−1.42**	**<0.001**	**0.33**
TRGI Total	1.33(0.59)	1.30 (0.61)	1.38(0.56)	−1.42	0.16	0.14
TRGI Global	1.35 (1.04)	1.25 (1.04)	1.50 (1.02)	**−2.47**	**0.01**	**0.24**
TRGI Distress	1.41 (0.77)	1.34 (0.75)	1.52 (0.77)	**−2.55**	**0.01**	**0.24**
TRGI Guilt Cognitions	1.35 (0.58)	1.34 (0.59)	1.37 (0.56)	−0.63	0.52	0.06
TRGI Hindsight Bias/Responsibility	0.87 (0.72)	0.85 (0.72)	0.90 (0.73)	−0.70	0.48	0.03
TRGI Wrongdoing	1.00 (0.82)	0.94 (0.81)	1.08 (0.84)	−1.78	0.08	0.17
TRGI Lack of Justification	3.22 (0.71)	3.20 (0.73)	3.25 (0.69)	−0.67	0.51	0.07

Bold values indicate statistically significant difference.

## Data Availability

The data presented in this study are available on request from the corresponding author due to privacy.
